# Bacterial Epimerization as a Route for Deoxynivalenol Detoxification: the Influence of Growth and Environmental Conditions

**DOI:** 10.3389/fmicb.2016.00572

**Published:** 2016-04-21

**Authors:** Jian Wei He, Yousef I. Hassan, Norma Perilla, Xiu-Zhen Li, Greg J. Boland, Ting Zhou

**Affiliations:** ^1^Guelph Research and Development Centre, Agriculture and Agri-Food Canada, GuelphON, Canada; ^2^School of Environmental Sciences, University of Guelph, GuelphON, Canada; ^3^Micotox Ltd.Bogota, Colombia

**Keywords:** deoxynivalenol, 3-*epi*-deoxynivalenol, transformation, epimerization, *Devosia*, growth conditions

## Abstract

Deoxynivalenol (DON) is a toxic secondary metabolite produced by several *Fusarium* species that infest wheat and corn. Food and feed contaminated with DON pose a health risk to both humans and livestock and form a major barrier for international trade. Microbial detoxification represents an alternative approach to the physical and chemical detoxification methods of DON-contaminated grains. The present study details the characterization of a novel bacterium, *Devosia mutans* 17-2-E-8, that is capable of transforming DON to a non-toxic stereoisomer, 3-*epi*-deoxynivalenol under aerobic conditions, mild temperature (25–30°C), and neutral pH. The biotransformation takes place in the presence of rich sources of organic nitrogen and carbon without the need of DON to be the sole carbon source. The process is enzymatic in nature and endures a high detoxification capacity (3 μg DON/h/10^8^ cells). The above conditions collectively suggest the possibility of utilizing the isolated bacterium as a feed treatment to address DON contamination under empirical field conditions.

## Introduction

Deoxynivalenol (DON, also known as vomitoxin) is a toxic secondary metabolite produced by several *Fusarium* species including *Fusarium graminearum* and *F. culmorum* (Audenaert et al., 2014). It is one of the most frequently detected mycotoxins in human foods and animal feeds worldwide (Lee and Ryu, 2015). DON presence within animal feed is connected with a myriad of immunological, reproductive, and developmental effects (Hassan et al., 2015d). The most characteristic toxicological symptoms of DON exposure in animals are feed refusal, body-weight loss and emesis (Pestka, 2010). DON is also a human health hazard that causes both acute and chronic effects associated with changes at the molecular and phosphoproteome levels (Wang et al., 2014). In plants, DON is believed to act as a virulence factor and was found essential for symptom development (Moretti et al., 2014). The toxicity of DON is conventionally attributed to its ability to inhibit ribosomal protein biosynthesis but recent studies have reported other novel mechanisms that further explain DON’s toxicological profile such as the ability to induce an oxidative-stress response and the involvement in intestinal barrier dysfunction (Hassan et al., 2015d).

The chemical nature of DON and its relative heat stability pose technical challenges to the management of DON-contaminated grains. Physical and chemical detoxification methods have been explored in the past and microbial detoxification represents an alternative approach that may provide a practical and effective solution for addressing DON-contaminated products (He and Zhou, 2010; McCormick, 2013). Several aerobic and anaerobic microorganisms selected from ruminants, swine, poultry, fish, and other agricultural commodities showed various DON transformation capabilities (Shima et al., 1997; Fuchs et al., 2000, 2002; Volkl et al., 2004; He and Zhou, 2010; Ikunaga et al., 2011; Ito et al., 2013). Despite the promising capabilities of these isolates, most of the reported bacteria require restrictive conditions for growth and DON bio-transformation, such as an anaerobic atmosphere (Fuchs et al., 2000, 2002) and/or the presence of DON as a sole carbon source (Ikunaga et al., 2011; Ito et al., 2013), which pose challenges for their empirical utilization.

The present study reports on the isolation and characterization of a unique bacterial strain capable of bio-transforming DON under aerobic conditions at mild temperatures. The bacterium was initially isolated from an alfalfa soil sample enriched with *F. graminearum* and moldy corn for several weeks. Microbiological and molecular characterization confirmed the affiliation of this bacterium with the *Devosia* genus.

The abrogation of toxicity of the biotransformation products was confirmed earlier using different human cell lines and mouse models (He et al., 2015a). The bacterium acts on the C-3 carbon in DON to epimerize the -OH group and produce 3*-epi*-DON (He et al., 2015b), eliminating the adverse toxicological effects associated with the consumption of DON in food/feed samples. It is hypothesized that the noticed isomeric changes influence 3*-epi*-DON ability to form hydrogen bonds within the peptidyl transferase center of ribosomes (A-site).

In addition, the role of different key growth and environmental factors such as pH, media formulations, nitrogen and carbon sources, and trace metals on the detoxification performance was elucidated confirming that DON epimerization takes place under a wide range of conditions and without the need for DON as the sole source of carbon in growth media.

## Materials and Methods

### Chemicals and Growth Media

Deoxynivalenol, glucose, sucrose, (NH_4_)_2_SO_4_, (NH_4_)_2_HPO_4_, K_2_HPO_4_, KH_2_PO_4_, MgSO_4_, K_2_SO_4_, FeSO_4_, MnSO_4_, NH_4_NO_3_7H_2_O were obtained from Sigma–Aldrich (Oakville, ON, Canada) or TripleBond (Guelph, ON, Canada). Methanol was obtained from Caledon Labs (Georgetown, ON, Canada). DIFCO Luria Bertani broth (LB), DIFCO Nutrient broth (NB), DIFCO Tryptic Soy Broth (TSB), DIFCO peptone, DIFCO tryptone, and DIFCO yeast extract were all purchased preparations.

A total of 11 different media formulations were evaluated and Corn meal broth (CMB) was used as the reference. To prepare corn meal broth without salts (CMB-WO-S): 40 g corn meal was soaked in 1 L water at 58°C for 4 h and allowed to stand for 2 h, followed by a filtration (Whatman No. 1, Whatman; Maidstone, Kent, UK). CMB refers to 1 L of CMB-WO-S supplemented with 3 g (NH_4_)_2_SO_4_, 1 g K_2_HPO_4_, 0.5 g MgSO_4_, 0.5 g K_2_SO_4_, 0.01 g FeSO_4_, 0.007 g MnSO_4_, and 5 g yeast extract. When 1.5% agar was added to CMB broth, it was referred to as Corn meal agar (CMA).

The following carbon sources were tested: glucose, sucrose, and corn starch. In addition, two categories of nitrogen sources were used: organic such as corn steep liquor, peptones, yeast extract, and urea; inorganic sources such as ammonium sulfate and ammonium nitrate. The concentrations of carbon and nitrogen sources were 10 g/L.

When minerals were added, the following formulation was used: 3 g (NH_4_)_2_SO_4_, 1 g K_2_HPO_4_, 0.5 g MgSO_4_, 0.5 g K_2_SO_4_, 0.01 g FeSO_4_, 0.007 g MnSO_4_, and 5 g yeast extract was incorporated per 1 L of final broth.

Other media compositions were: Yeast + glucose (YG): 1 L water with 5 g yeast and 10 g glucose. BYE: 1 L water with 0.5 g of NH_4_NO_3_, 0.2 g of yeast extract, 50 mg of H_3_BO_4_, 40 mg of MnSO_4_⋅4H_2_O, 20 mg of (NH_4_)_6_Mo_7_O_24_, 4 mg of CuSO_4_⋅5H_2_O, 4 mg of CoC_l6_⋅6H_2_O and 5 mM potassium phosphate buffer (adjusted to pH 7.0 with NaOH) (Shima et al., 1997). Minimal medium (MM): 1 L medium contained 10 g sucrose, 2.5 g K_2_HPO_4_, 2.5 g KH_2_PO_4_, 1 g (NH_4_)_2_HPO_4_, 0.2 g MgSO_4_⋅7H_2_O, 0.01 g FeSO_4_, and 0.007 g MnSO_4_. MM + yeast medium (MMY): MM medium with 0.5% yeast extract. Rice medium (RM) was prepared in a similar fashion to CMB. Corn meal broth + peptone + dextrose medium (CMBPD): CMB with 2% peptones and 2% dextrose. MM + peptones + tryptone medium (MMPT): MM medium with 1% peptones and 1% tryptone.

### Isolation of the Bacterium 17-2-E-8

In 2007 and during the characterization of microorganisms isolated from an alfalfa soil sample that was enriched with *F. graminearum*-infested moldy corn for 6 weeks, a bacterial isolate 17-2-E-8 was selected on CMA incubated at 28°C for 72 h. In essence, soil suspensions were serially diluted using CMB medium and both DON reduction and bacterial growth were monitored. For DON reduction, 100 μL of each serial dilution was sub-cultured with 900 μL CMB containing DON (100 μL of 1000 μg/mL DON) at 28°C on a rotary shaker at 200 rpm for 72 h. Cultures were analyzed later as described below.

For bacterial growth, 100 μL of each serial dilution were cultivated on a CMA plate and incubated at 28°C. After 48–72 h incubation, the total number of CFU was calculated. Serial dilutions showing the lowest number of microorganisms yet exhibiting a reduction in DON concentrations were selected to be further pursued. The above procedure was repeated eight times and single colonies were picked from plates corresponding to the highest dilution which still showed a reduction in DON concentrations. These colonies were sub-cultured in CMB and their activities were evaluated individually using the methods described earlier. Ultimately, a bacterium showing transparent to white-colored colonies with strong DON-bio-transformation capabilities was isolated.

To follow how the cell numbers of the purified bacterium correlated with its ability to reduce DON concentrations, a tube containing 10 mL of CMB medium was inoculated with a loop of bacterial cell suspension (1 μL). The culture was incubated aerobically at 28°C with shaking at 200 rpm. This culture was monitored for bacterial growth, decrease in DON concentrations, and formation of the major compound resulting from DON bio-transformation, 3-*epi*-DON (He et al., 2015b). The microbial growth was determined using serial dilution plating methods while DON and *3-epi*-DON concentrations were tracked using high performance liquid chromatography (HPLC) (as described later).

### The Influence of Environmental Factors on the Bio-transformation of DON by the Isolate 17-2-E-8

To follow how different environmental factors affect the growth of isolate 17-2-E-8 and its ability to reduce DON concentrations, 10 mL CMB medium were inoculated with a loop of 17-2-E-8 bacterial cells (1 μL). The culture was incubated at 28°C for 72 h with continuous shaking at 200 rpm. This culture was then adjusted to a cell concentration of 1 × 10^6^ CFU/mL using CMB based on optical density (OD_600_) and served as a seed culture.

To test the effect of aerobic/anaerobic conditions, shaking, and culture media ingredients on the growth and DON reduction by isolate 17-2-E-8, testing mixtures were prepared by adding 100 μL bacterial culture from the above seed culture at a cell concentration of 1 × 10^6^ CFU/mL, and 100 μL of DON solution containing 1000 μg/mL, into 800 μL of the respective media, resulting in the final bacterial concentration of 1 × 10^5^ CFU/mL and final DON concentration at 100 μg/mL. These mixtures were incubated at 28°C for 72 h under aerobic conditions on a rotary shaker at 200 rpm, and also under anaerobic conditions (5% H_2_ and 10% CO_2_ balance N_2_) at 28°C with hand-mixing approximately every 6 h.

The temperature effect on the bacterial growth and DON epimerization was tested in 1.5 mL tubes with 1 mL CMB medium containing isolate 17-2-E-8 (∼1 × 10^5^ CFU/mL) and 100 μg/mL DON. The tubes were incubated at 5, 10, 15, 20, 25, 30, 35, and 40°C, respectively, on a rotary shaker at 200 rpm. In a similar fashion, pH effect on the bacterial growth and reduction of DON was elucidated by preparing aliquots of CMB with adjusted pH values of 3, 4, 5, 6, 7, 8, 9, and 10, respectively, using NaOH or HCl 1 mol/L. After inoculation using the above seed culture, test tubes were incubated at 28°C and 200 rpm shaking under aerobic conditions for 48 h. The samples were extracted and analyzed as described later.

### Mechanism of DON Bio-transformation by Isolate 17-2-E-8

The interactions of DON with the inactivating microorganisms have been reported to take different forms spanning the binding of bacterial cells to enzymatic bio-transformations (Volkl et al., 2004; Awad et al., 2010; Islam et al., 2012; Hassan and Bullerman, 2013). In order to narrow down the nature of the observed interactions between DON and isolate 17-2-E-8, we tested the ability of heat and acid-inactivated cells to reduce DON levels in growth medium and tracked the accumulation of 3-*epi*-DON in broth at the same time.

A 30 mL dense culture (OD_600_ > 2) of isolate 17-2-E-8 was split to three equal parts. The first part (10 mL) represented the living cells while the two other parts (10 mL each) represented the heat and acid-inactivated cells. The heat-inactivated cells were subjected to autoclaving (15 min. at 121^o^C) while the acid-inactivated cells were pelleted by centrifugation and treated with 1N HCl for 2 h. After a washing step with sterile phosphate buffer saline (PBS), acid-inactivated cells were re-suspended to the original volume (10 mL). 0.5 mL of each of the above treatments was mixed with 0.5 mL of fresh LB broth containing 50 μg/mL DON (to yield a final DON concentration of 25 μg/mL). Final tubes (in triplicates) were incubated at 28° overnight at 120 rpm. Actively growing cells of *Devosia riboflavina* Strain IFO13584 were included as a negative control. All the samples were analyzed for DON reduction/epimerization as described below.

### Analysis of DON and 3*-epi-*DON

To each 500 μL bacterial culture, 500 μL methanol (analytical grade) was added. The mixture was allowed to shake for 2 h and filtered through a 0.45 μm polyvinylidine fluoride (PVDF) syringe filter (Whatman; Maidstone, Kent, UK) before analyzing by HPLC. DON and 3-*epi*-DON separation was achieved according to He et al. (2015b) using an Agilent Technologies 1100 Series HPLC system equipped with a Luna C18 column (150 × 4.6 mm, 5 μm) (Phenomenex, Torrance, CA,USA). The binary mobile phase consisted of solvent A (methanol) and solvent B (water) and the gradient program started at 22% A, increased linearly to 41% A at 5 min, 100% A at 7 min, held at 100% A from 7 to 9 min, and returned to 22% A at 11 min. There was a 2 min post-run column re-conditioning phase under the starting conditions. The flow rate was 1.0 mL/min and the detector was set at 218 nm. Identification of DON and 3-*epi*-DON (He et al., 2015b) was achieved by comparing the retention times and UV-Vis spectra. DON/3-*epi*-DON concentrations were quantified based on reference to a calibration curve of DON/3*-epi-*DON standards (He et al., 2015a,b).

### Microbiological Characterization

The cell morphology of the isolate was observed using scanning electron microscopy (SEM) and transmission electron microscopy (TEM) for cells grown at 28°C in TSB. The SEM analysis was conducted in the Electron Microscope Lab, Department of Food Science, University of Guelph; while image acquisition with TEM was conducted at the Guelph Regional Integrated Imaging Facility, Department of Molecular, and Cell Biology, University of Guelph.

The motility of isolate 17-2-E-8 was tested by stabbing 15 mL slants containing either motility test medium (beef extract 3 g, peptone 10 g, sodium chloride 5 g, and agar 4 g/L) or soft CMA (CMB prepared as described above and supplemented with 4 g/L agar). Slants were kept at 28–30°C and evaluated after 36–48 h of incubation. Disk diffusion antimicrobial susceptibility tests were performed at 28–30°C for 24 h either on Mueller Hinton or LB agar plates using the following antibiotic disks: streptomycin (10 μg), chloramphenicol (30 μg), tetracycline (30 μg), penicillin G (10 IU), and kanamycin (30 μg).

The gas chromatographic analysis of fatty acid methyl esters (GC-FAME) was conducted twice independently. Isolate 17-2-E-8 cells were either grown in LB broth for 7 days before inoculating 500 mL (LB broth) with the actively growing cells and incubating the flask on an orbital shaker (120 rpm) at room temperature (23–25°C) for 48 h. The lyophilized cells were sent later to the Identification Services at Deutsche Sammlung von Mikroorganismen und Zellkulturen (DSMZ) (Braunschweig, Germany) for analysis. Alternatively, the cells were streaked on Tryptic Soy Agar (TSA) and grown for 5 days at 28°C and the cells were then analyzed by the University of Guelph, Laboratory Services (Guelph, ON, Canada). The obtained chromatographic results (patterns and recognition) were compared with the Sherlock library (Version 4.5; MIDI Inc.; Nework, DE, USA; 2002).

Respiratory quinones were analyzed at DSMZ on freeze-dried cells using the two stage method described by Tindall (1990a,b).

The ability of isolate 17-2-E-8 to oxidize different carbon sources was investigated using BIOLOG bacterial identification system (BIOLOG, Hayward, CA, USA). Briefly, the bacterium was grown on Biolog Universal Growth agar plates supplemented with 5% sheep blood, harvested, and re-suspended in Gram Negative/Gram Positive (GN/GP) inoculating fluid. Suspensions (150 μL) were pipetted into each well of the GN2 MicroPlate and incubated at 30°C overnight. Carbon and amino acids utilization patterns were assessed and tolerance toward lactic acid, the reducing power, and sensitivity against a large array of chemical compounds were determined. Isolate 17-2-E-8 metabolic fingerprint was compared with the MicroLog database (BIOLOG).

A Matrix Assisted Laser Desorption/Ionization-Time of Flight (MALDI-TOF) bacterial identification based on the mass spectrometry signature of ribosomal proteins (Chalupova et al., 2014) was completed at MIDI Labs, Inc. (Newark, DE, USA) to match 17-2-E-8 to the closest bacterial species.

### 16S rRNA Gene Sequencing and Phylogenetic Analysis

The 16S rRNA gene sequence of 17-2-E-8 was obtained by preparing genomic DNA using Puregene Yeast/Bacteria Kit B (Qiagen, Toronto, ON, Canada) from a dense overnight culture. Primers fD1 and rD1 (Bresler et al., 2000) were used to amplify the 1.5 kb gene with 45° annealing temperature. Gel-purified polymerase chain reaction (PCR) products were then used in sequencing reactions using the same amplification primers. The almost complete (1421 bp) retrieved-sequence was searched against the entire collection of 16S ribosomal RNA sequences (bacteria and archaea) within NCBI GenBank to determine the most closely related genus/species (Zhang et al., 2000). For the reconstruction of phylogenetic trees; MEGA version 6.0 software package was used after multiple sequence alignments/comparisons using CLUSTAL_W (Gap opening penalty = 15, Gap extension penalty = 6.66). Trees were reconstructed using neighbor-joining (NJ) and maximum-parsimony (MP) methods with 1000 bootstrapping, Kimura2-parameter (for NJ), and subtree pruning/re-grafting (for MP) models. DNASTAR lasergene 8 software package (DNASTAR, Madison, WI, USA) was also used to confirm tree outcomes. The default settings of MegAlign were: gap penalty = 15.00, gap length penalty = 6.66, delay divergent seqs (%) = 30 and DNA transition weight 0.50. Bootstrap trials were 1000 with seed = 111 (Burland, 2000). Sequence similarity was calculated using the SIAS server^1^. The DNA G + C content of isolate 17-2-E-8 was calculated from a *de novo* genome assembly conducted and deposited recently (Hassan et al., 2014).

### Next-Generation Whole-Genome Sequencing and Species Comparisons

Recent advancements in next-generation sequencing platforms have added a new dimension for bacterial isolates comparisons (Hassan et al., 2015c). Using the advantages of such a technique we compared the genome sequence of isolate 17-2-E-8 with other available type strains. The *de novo* sequencing of the entire genome of isolate 17-2-E-8 was accomplished as reported earlier (Hassan et al., 2014). Other type strains representing different *Devosia* species were obtained from DMSZ culture collection (Braunschweig, Germany) and the whole-genome sequencing of these strains was conducted as reported (Hassan et al., 2014, 2015a,b).

Pair-wise comparisons of multiple *Devosia* type-strains genomes were conducted using BRIG (Alikhan et al., 2011) with the default parameters. The entire genome of *Devosia* 17-2-E-8 was aligned with *D. geojensis* (DSM19414), *D. psychrophila* (DSM22950), *D. chinhatensis* (DSM24953), *D. soli* (DSM17780), *D. limi* (DSM17137), *D. epidermidihirudinis* (DSM25750), and *D. riboflavina* (IFO13584) genomes.

### Statistical Analysis

For DON concentrations and bacterial cell numbers, samples were analyzed in triplicate and the means were determined. The relevant reduction of DON was calculated as the following: Reduction in DON concentration (%) = (C_DON_ _added_–C_DON_ _residual_)/C_DON_ _added_ × 100. Data were analyzed using SAS (SAS for Windows, Version 9.1, SAS institute, Cary, NC, USA), SigmaStat Version 3.11 (Systat Software, Point Richmond, CA, USA), or Sigmaplot 12.5 (Systat Software Inc). Data were tested for normality using the Kolmogorov–Smirnov method and equal variance (*P* value to reject was set for 0.05). Multiple group comparisons of normally distributed data were conducted by One Way Analysis of Variance (One Way ANOVA), followed by *post hoc* pairwise comparisons using the Holm–Sidak test or Fisher’s protected least significant difference (PLSD). Multiple group comparisons of non-parametric data were conducted using the Kruskal–Wallis ANOVA on Ranks, followed by *post hoc* pairwise comparisons using the Dunn’s method.

## Results

### Reduction of DON Concentrations Correlated with the Growth of Bacterial Isolate 17-2-E-8

**FIGURE 1:**
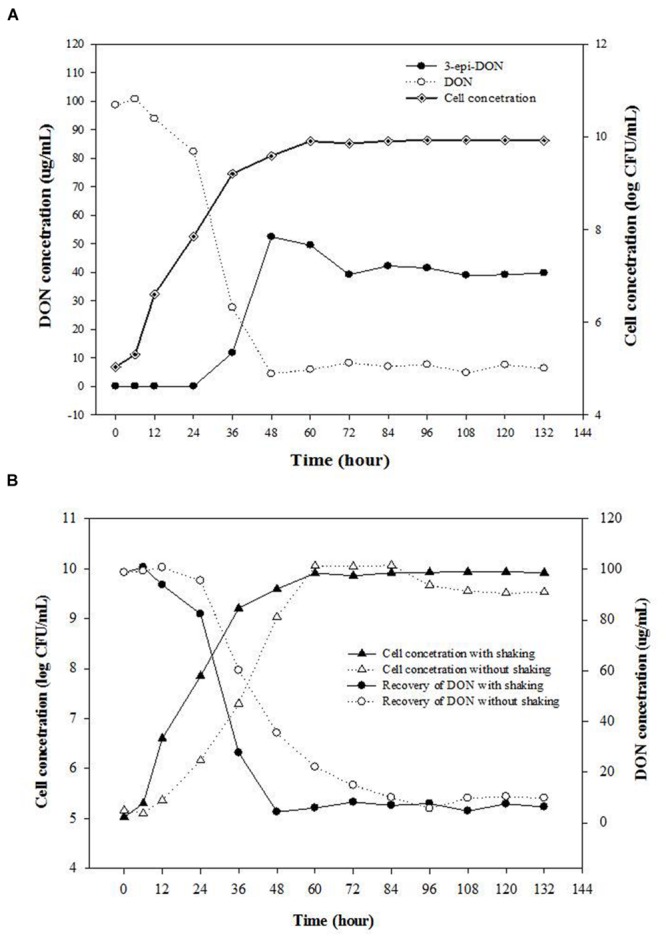
**Isolate 17-2-E-8 is capable of transforming DON into 3*-epi-*DON as a major product under aerobic conditions. (A)** The effect of incubation times on the bacterial growth and DON concentrations in corn meal broth (CMB) cultures containing 100 μg/mL DON and inoculated with bacterium 17-2-E-8. The cultures were kept shaking (200 rpm) at 28°C. The PLSD_(0.05)_ of the tests for concentration of DON and 3*-epi*-DON, and cell concentration were 7.6 and 0.38, respectively. **(B)** The effects of shaking on bacterial cell numbers of isolate 17-2-E-8 and reduction of DON levels in CMB cultures containing 100 μg/mL DON. The PLSD_(0.05)_ of the tests for DON concentration and viable cell numbers were 8.1 and 0.38, respectively.

The effect of incubation times on the bacterial growth and DON concentrations in CMB cultures (containing 100 μg/mL DON) was tested. A coincidence of increasing cell numbers of isolate 17-2-E-8 and decreasing DON concentrations was observed (**Figure 1A**). The maximum increase in bacterial cells and decrease in DON concentrations was observed within 72 h of inoculating CMB medium. During the bacterium exponential growth (6–36 h), a substantial decrease in DON concentrations within growth medium was observed. By the time the bacterium reached the stationary growth phase (almost 48 h after inoculation), DON concentrations fell drastically to low levels (5 μg/mL). As reported earlier, the bacterium 17-2-E-8 transforms DON to 3-e*pi*-DON as the major metabolite under aerobic conditions (He et al., 2015b). A matched accumulation in 3*-epi-*DON within growth media developed in parallel with DON disappearance from growth medium. As shown by (**Figure 1A**), 3-*epi*-DON levels kept accumulating within test tubes in parallel to DON reduction until reaching the maximum levels noted around 60–72 h of incubation. Thereafter, 3-*epi*-DON levels did not change even with extended incubation times (up to 132 h). The initial trials indicated that isolate 17-2-E-8 had the capacity to detoxify DON at rates close to 3 μg/h/10^8^ cells (**Figure 1A**) within the exponential growth phase (24–36 h).

### Bio-transformation of DON Proceeded under Aerobic Conditions

The effects of shaking/aerobic/non-aerobic conditions on bacterial cell numbers of isolate 17-2-E-8 and reduction of DON levels in CMB cultures were checked. No increase in bacterial cell numbers or any associated reduction in DON concentrations was observed when isolate 17-2-E-8 was incubated for 72 h in CMB medium under anaerobic conditions (data not shown). However, increases in bacterial cell numbers and reduction in DON concentrations were observed when isolate 17-2-E-8 was incubated for 72 h in CMB medium under aerobic conditions (**Figure 1B**). The rotary action (200 rpm) of the orbital shaker impacted the cell’s growth, assumingly by altering the oxygen supply, and at the same time affected the DON-transformation activities of isolate 17-2-E-8. With continuous shaking, maximum cell numbers and maximum DON reduction levels were observed around 48 h of the incubation period (**Figure 1B**).

### Growth of Isolate 17-2-E-8 and DON Bio-transformation Proceeded Favorably at Mild Temperature

A wide range of temperatures spanning 5–40° were selected to check their effect on supporting isolate 17-2-E-8 growth and DON epimerization. The effect of growth temperatures on DON levels in CMB cultures spiked with 100 μg/mL DON and inoculated with isolate 17-2-E-8 are shown in **Table 1**.

**Table 1:** Growth and DON biotransformation activities of isolate 17-2-E-8 at selected cultivation temperatures.

Temperature (°C)	Growth (CFU/ml)^1,2^	DON biotransformation (%)^2^
5	2.6 × 10^6 a^	0.8^a^
10	1.2 × 10^7 a^	0.8^a^
15	2.8 × 10^6 a^	1.2^a^
20	1.0 × 10^9 b^	82.0^b^
25	1.8 × 10^9 c^	85.2^b^
30	2.5 × 10^9 d^	88.0^b^
35	2.4 × 10^9 d^	88.5^b^
40	1.6 × 10^9 bc^	1.3^a^

*^1^Determined after 48 h in CMB (with minerals) at pH 6.9.*

*^2^Values in the same column with different superscripts differ significantly according to Tukey’s multiple range test (*p* = 0.05).*

As shown, the highest growth was observed within the 25–35° range after 48 h incubation. Isolate 17-2-E-8 cell numbers were significantly higher in this range compared to lower or higher temperatures. On the other side, the bio-transformation of DON continued at maximum velocity within the 20–35° range (**Table 1**). Unfavorable temperatures (as low as 15° or as high as 40°) limited DON bio-transformation capabilities of isolate 17-2-E-8 cells. The influence of low/high temperatures was more drastic on the epimerization function in comparison to cell growth. For example, the cells reached 1.6 × 10^9^ CFU/mL at 40° (**Table 1**), but DON transformation merely exceeded a level of 1.3%.

A follow-up experiment (data not shown) indicated that incubation at 28°C demonstrated the same bio-transformation efficiency as 25°C, whereas the required time was shorter. Therefore, a temperature of 28°C was selected as the optimum temperature for the following experiments.

### Neutral pH Values Maximized Isolate 17-2-E-8 Bio-transformation Capacity

pH is considered as an influential factor that affects bacterial growth/function. The effect of this factor was assessed by growing isolate 17-2-E-8 in CMB with adjusted pH values and determining growth and DON biotransformation patterns. **Table 2** shows how different pH values affected both isolate 17-2-E-8 growth and DON bio-transformation capacity. The total number of viable cells indicated that isolate 17-2-E-8 increased in numbers as the acidity decreased within the media and pH values came closer toward neutrality (pH = 7). Shifting toward alkalinity (pH = 9–10) reversed the observed increase in cell numbers causing a significant inhibitory effect but to a milder degree compared to the acidic pH range. In contrast to the gradual decline of the total number of viable cells observed due to the deviation from the neutral pH range, DON epimerization function was affected substantially in comparison. The most noticeable reductions in DON concentrations were only observed at pH values ranging from 6 to 8 in general and around pH = 7 more specifically (**Table 2**).

**Table 2:** The growth and biotransformation of DON^1^ by isolate 17-2-E-8 at selected initial media pH values.

Initial pH	Number of viable cells (log CFU/mL)^1,2^	DON concentrations recovered from cultures (%)^2^
3	6.41^a^	98.0^d^
4	6.43^a^	98.4^d^
5	7.60^b^	97.7^d^
6	9.04^d^	22.5^b^
7	9.00^d^	4.9^a^
8	8.04^c^	62.2^c^
9	7.94^bc^	92.5^d^
10	7.98^bc^	98.6^d^

*^1^Determined after 48 h bacterial cultivation in corn meal broth (CMB) at 28°C and 200 rpm.*

*^2^Values in the same column with different superscripts differ significantly according to Tukey’s multiple range test (*p* = 0.05).*

### Media Affected the Growth of Isolate 17-2-E-8 and DON Bio-transformation

Media composition had a strong influence on DON-transformation capacity of isolate 17-2-E-8. For example, both CMB and YG media supported the highest accumulation of 3-*epi*-DON with 49.2 and 89.3 μg/mL, respectively, and the lowest DON residual with 4.6 μg/mL and below the detection limit (BDL) concentrations, respectively (**Table 3**). In contrast, MMPT medium did not show any support for the epimerization function of DON (**Table 3**).

**Table 3:** Residual DON and its bio-transformation products after culturing isolate 17-2-E-8 in different media preparations supplemented with 100 μg/mL DON, at 28°C with continuous shaking at 200 rpm for 72 h.

Medium	3-epi-DON (μg/mL)^1^	DON (μg/mL)^1^
Yeast+Glucose (YG)	89.3 ^h^	BDL ^a∗^
CMB	49.2 ^e^	4.6 ^a^
BYE	58.0 ^f^	14.6 ^b^
Nutrient broth	72.2 ^g^	17.7 ^b^
MMY	24.7 ^c^	30.5 ^c^
Luria Bertani (LB)	34.5 ^d^	31.0 ^c^
CMB-WO-S	37.9 ^d^	32.4 ^cd^
TSB	39.2 ^d^	37.0 ^d^
Rice medium (RM)	15.8 ^b^	76.4 ^e^
CMBPD	22.2 ^c^	81.1 ^e^
MMPT	BDL ^a∗^	93.9 ^f^

*^1^Values in the same column with different superscripts differ significantly according to Tukey’s multiple range test (*p* = 0.05).*

*^∗^ BDL, below the detection level.*

Our observation related to the influence of different media compositions on DON biotransformation rates of isolate 17-2-E-8 (**Table 3**) triggered a more systematic approach to understand the influence of different carbon and nitrogen sources in addition to the role of minerals. As indicated in **Table 4**, sources rich in complex organic nitrogen (particularly peptones and yeast extract) supported isolate 17-2-E-8 growth and maximum DON transformation with levels close to 99% of total DON concentration (**Table 4**) while inorganic nitrogen sources (such as ammonium sulfate, and ammonium nitrate) were limiting in supporting the biotransformation capacity (1–22%). This might be attributed to the role of organic nitrogen in supporting protein biosynthesis (essential and non-essential amino acids) needed for enzyme functionality/activity.

**Table 4:** The effect of carbon, nitrogen, and minerals sources on growth and DON biotransformation activity of Devosia mutans Strain 17-2-E-8^1^.

Nutrient (10 g/l)		Initial pH^2^		Growth (CFU/ml)^4^		DON Biotransformation (%)^4,5^	
		Without minerals	Minerals added^3^	Without minerals	Minerals added	Without minerals	Minerals added
Carbon	Glucose	5.0	7.5	1.6 × 10^7 a^	1.2 × 10^7 a^	0.8 ^a^	1.0 ^a^
	Sucrose	6.3	7.9	8.6 × 10^6 a^	4.0 × 10^7 a^	1.0 ^a^	8.3 ^a^
	Corn starch	5.2	7.7	2.8 × 10^6 a^	9.9 × 10^6 a^	0.7 ^a^	48.0 ^ab^
Nitrogen (Organic)	Corn steep liquor	4.5	6.7	2.9 × 10^9 ab^	3.5 × 10^9 b^	17.1^a^	99.5 ^b^
	Peptones	7.2	7.6	1.5 × 10^9 ab^	2.5 × 10^9 b^	49.3 ^ab^	99.2 ^b^
	Yeast	6.0	7.2	5.3 × 10^9 b^	8.7 × 10^9 c^	87.7 ^b^	84.4 ^b^
	Urea	9.3	9.0	2.8 × 10^6 a^	2.8 × 10^6 a^	1.0 ^a^	1.0 ^a^
Nitrogen (Inorganic)	Ammonium sulfate	4.4	6.9	2.9 × 10^6 a^	3.0 × 10^6 a^	1.4 ^a^	22.0 ^a^
	Ammonium nitrate	4.9	6.6	6.9 × 10^6a^	3.0 × 10^6 a^	1.0 ^a^	18.6 ^a^
Control	Corn meal Broth (control)		6.9		7.7 × 10^9 b^		97.8

*^1^Determined after 72 h in shaken culture (200 rpm) at 28°C.*

*^2^Values of media pH are shown.*

*^3^Minerals (per liter): K_2_HPO_4_, 1.0 g; MgSO_4_, 0.5 g; K_2_SO_4_, 0.5g; FeSO_4_, 0.1 g, and 0.07 MnSO_4_, 0.07 g.*

*^4^Values in the same column with different superscripts differ significantly according to Tukey’s multiple range test (*p* < 0.05).*

*^5^The percentage of DON bio-transformation was estimated by subtracting the remaining DON after incubation from the initial concentration, multiplied × 100.*

The role of minerals, mainly as co-factors in the enzymatic reactions responsible for epimerization of DON to 3-*epi*-DON is illustrated by the effect of incorporating mineral mixtures on growth and DON conversion rates (**Table 4**). While minerals did not influence the growth rate within corn steep liquor or peptone broth (with similar bacterial counts within the 1.5–3.5 × 10^9^ range), it did significantly influence the bio-transformation rates enhancing them from 0.7 to 48% in corn steep liquor and from 17 to 99% in peptones broth, respectively (**Table 4**). This effect was also observed in the media supplemented with inorganic nitrogen where DON biotransformation was efficiently enhanced (independent from bacterial growth) from 1.0–1.4 to 18–22% range in both ammonium sulfate and ammonium nitrate broths.

### Bio-transformation of DON by Isolate 17-2-E-8 Was Enzymatic in Nature

**FIGURE 2:**
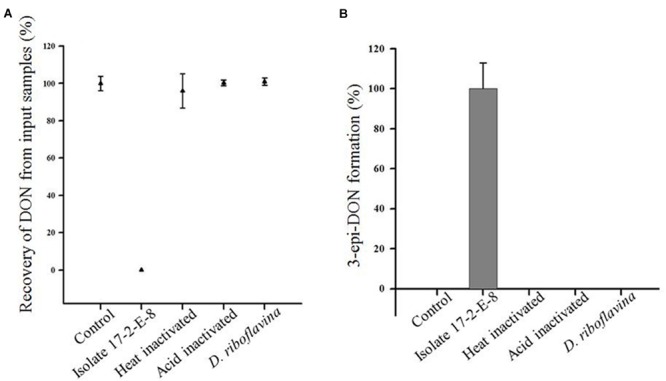
**Recovery of DON **(A)** and production of 3-epi-DON **(B)** by viable cells of isolate 17-2-E-8.** Neither heat nor acid-inactivated cells showed the same tendency. The accumulation of *3-epi-*DON was a characteristic of isolate 17-2-E-8 compared to *Devosia riboflavina* Strain IFO13584.

In order to further track the mechanistic nature of DON reduction by isolate 17-2-E-8; both viable and inactivated cells (heat/acid) were tested separately. Cells of *D. riboflavina* Strain IFO13584 were included as a negative control. **Figure 2** shows that DON concentrations were reduced to an undetectable levels only when incubated overnight with viable cells (**Figure 2A**). This reduction in DON was accompanied by the accumulation of 3-*epi*-DON in the culture media (**Figure 2B**). At the same time, none of the heat or acid-inactivated cells led to any detectable decrease in DON levels or the appearance of 3-*epi*-DON within growth media. As expected, the viable cells of *D. riboflavina* Strain IFO13584 were not able to bio-transform DON to 3-*epi*-DON nor influence DON concentrations within the test tubes (**Figure 2**). Collectively, these results support the notion of an enzyme-carried function.

### Microbiological Characterization Confirmed the Affiliation with the *Devosia* Genus

Our earlier observations indicated that the isolate 17-2-E-8 belongs to the genus *Devosia* possibly representing a new species (He et al., 2015a). A further phenotypical characterization coupled with more genomic data was needed to confirm these observations.

**FIGURE 3:**
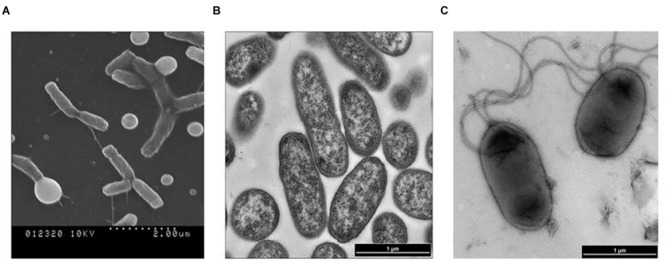
**Scanning electron microscopy (SEM) imaging of *Devosia mutans* 17-2-E-8 **(A)** and transmission electron microscopy (TEM) images **(B,C)** of isolate 17-2-E-8**.

The acquired SEM and TEM images, in addition to Gram-staining patterns, clearly indicated that cells of isolate 17-2-E-8 had an oval to rod-shaped morphology (**Figure 3A**) that stained negative with Gram-staining. Cell dimensions were 1–1.8 μm in length and 0.4–0.8 μm in width (**Figure 3B**). The bacterial cells were able to form 1–4 polar flagella (**Figure 3C**). These cells formed circular, raised, and transparent to white-colored colonies with smooth edges when grown on CMA, TSA, and nutrient agar. The colonies seemed to lack the shiny reflection noticed with *D. riboflavina* (IFO 13584, J.W. Foster 4R3337) colonies growing on the same media (**Table 5**). Using *D. riboflavin*a (Foster) Nakagawa et al. (1996) (ATCC 9526) as a reference/control, isolate 17-2-E-8 was resistant to kanamycin and chloramphenicol but susceptible to penicillin G and tetracycline. It showed intermediate sensitivity toward streptomycin. Isolate 17-2-E-8 was motile in both motility/soft CM slants.

**Table 5:** Differential phenotypic characteristics of *Devosia mutans* 17-2-E-8 in comparison to other *Devosia* species.

Characteristic	*Devosia* 17-2-E-8	*D. riboflavin* DSM 7230^T^	*D. insulae* DS-56^T^	*D. soli* GH2-10^T^	*D. neptuniae* J1^T^	*D. chinhatensis* IPL18^T^	*D. epidermidihirudinis* E84^T^	*D. submarina* KMM 9415^T^	*D. pacifica* NH131^T^
Colony color	Transparent to white^a^	Cream, shiny^a^	Ivory^a^	Light beige^a^	Pearl white^a^	Cream^a^	Beige, shiny^a^	orange–reddish^a^	White^a^
Cell shape	Ovals to rods	Rods	Ovals or rods	Rods	Rods	Rods	Rods	Rods	Short rods
Motility	+	+	+	ND	+	+	-	+	_+_
Flagellum	1–4 polar	several polar	1 polar	ND	1 polar	1 polar	ND	2–7 polar, bipolar and lateral	1 polar
Glucose	+	+	-	-	+	+	+	+	+
L-Arabinose	+	+	-	-	+	+	+	+	-
D-Mannitol	-	+	-	-	+	+	+	+	-
Mannose	+	+	-	-	+	+	+	+	+
Maltose	+	+	-	-	+	+	+	+	_+_
Galactose	+	+	ND	ND	-	ND	ND	-	-
Sucrose	(+)	-	ND	ND	ND	ND	ND	-	-
Lactose	+	+	ND	ND	ND	ND	ND	-	+
*myo*-Inositol	-	(+)	ND	ND	ND	ND	ND	-	-
L-Fucose	+	ND	ND	ND	ND	ND	ND	-	+

*Species: 1, *D. mutans* 17-2-E-8; 2, *D. riboflavin* DSM 7230^T^; 3, *D. insulae* DS-56^T^; 4, *D. soli* GH2-10^T^; 5, *D. neptuniae* J1^T^; 6, *D. chinhatensis* IPL18^T^; 7, *D. epidermidihirudinis* E84^T^; 8, *D. submarina* KMM 9415^T^; 9, *D. pacifica* NH131^T^. +, Positive; -, negative; (+), weakly positive; ND, no data available. ^a^All strains are non-spore-forming.*

**FIGURE 4:**
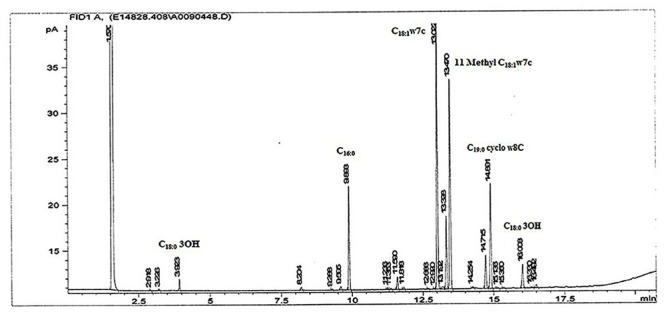
**The fatty acid methyl ester profile of isolate 17-2-E-8 using GC-FAME.** Peaks were identified on the basis of their retention times using BAME (bacterial methyl ester) standards.

**FIGURE 5:**
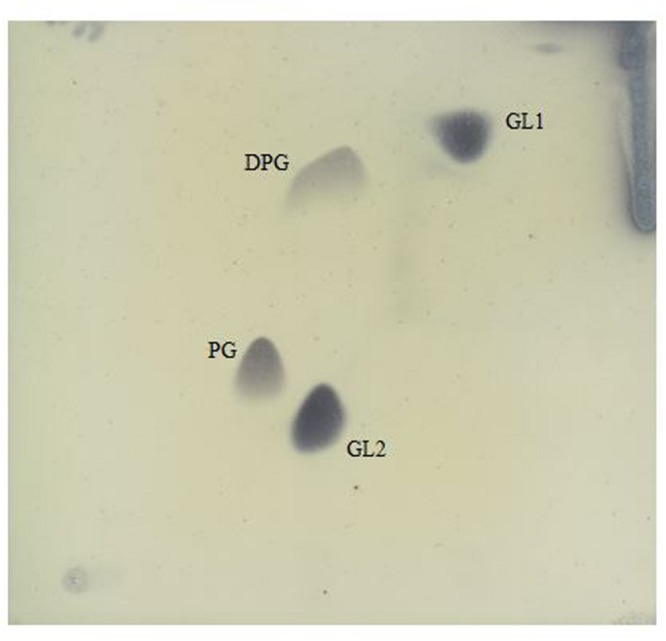
**Polar lipids of isolate 17-2-E-8 as separated by two dimensional silica gel thin layer chromatography.** The first direction is developed in chloroform:methanol:water (65:25:4, v/v/v), and the second in chloroform:methanol:aceticacid:water (80:12:15:4, v/v/v/v). Four spots were detected reflecting glycolipids (GL1 and GL2), diphosphatidylglycerol (DPG), and phosphatidylglycerol (PG) fractions.

The obtained fatty acid profile of 17-2-E-8 is shown in **Figure 4**, and was mainly composed of C_16:0_ (11.64%), C_18:0_ (8.68%), C_18:1_ω7c (31.78%), C_10:0_ 3-OH (1.0%), C_18:0_ 3-OH (3.04%), 11-methyl C_18:1_ω7c (26.26%), C_19:0_cycloω8c (13.19%), and C_17:0_ (1.5%). This profile was close in nature to several members of the *Devosia* genus (**Table 6**). While we had some concerns about the elevated levels of 11-methyl C_18:1_ω7c and C_19:0_cycloω8c in the final profile, the identical values present in two independent analyses (25.1%, 13.3% in the first analysis and 26.26%, 13.19% in the second analysis for 11-methyl C_18:1_ω7c and C_19:0_cycloω8c, respectively) confirmed the validity of the obtained profile in this study, despite the slightly differing growth conditions/times. The predominant isoprenoid quinone was identified as Q10 (100%) while Q11 was not detectable in our sample. This is in accordance with most validly reported *Devosia* species where Q10 is the principle detected ubiquinone except for *D. insulae* (Yoon et al., 2007). The analysis of polar lipids reflected the presence of phosphatidylglycerol and di-phosphatidylglycerol in addition to two unidentified glycolipid bands labeled as GL1 and GL2 (**Figure 5**).

**Table 6:** Cellular fatty acid compositions (%) of strain 17-2-E-8 and type strains of species of the genus *Devosia.*

Fatty acid	*Devosia* 17-2-E-8	*D. riboflavin* DSM 7230^T^	*D. insulae*DS-56^T^	*D. soli*GH2-10^T^	*D. neptuniae* J1^T^	*D. chinhatensis* IPL18^T^	*D. epidermidihirudinis* E84^T^	*D. submarina* KMM 9415^T^	*D. pacifica* NH131^T^
C_16:0_	11.64	22.0	12.7	12.7	25.6	20.6	23.7	8.77	7.4
C1_8:0_	8.68	3.8	3.2	3.0	6.7	8.1	6.8	4.5	12.2
C_18:1_ω7c	31.78	44.7	22.5	67.2	21.6	52.4	5.4	64.7	51.1
C_10:0_ 3-OH	1	1.7	–	3.5	0.7	–	–	1.5	–
C_18:0_ 3-OH	3.04	0.4	–	5.2	5.5	2.2	3.6	5.2	–
11-Methyl C_18:1_ω7c	26.26	21.8	30.2	5.8	27.0	10.3	33.8	5.6	21.6
C_19:0_ ω8c cyclo	13.19	1.6	1.1	–	7.8	–	24.9	–	1.7
Summed feature 3^∗^	0.42	2.4	0.6	2.8	1.9	–	–	–	0.7

Species: 1, *D. mutans* 17-2-E-8^;^ 2, *D. riboflavin* DSM 7230^T^; 3, *D. insulae* DS-56^T^; 4, *D. soli* GH2-10^T^; 5, *D. neptuniae* J1^T^; 6, *D. chinhatensis* IPL18^T^; 7, *D. epidermidihirudinis* E84^T^; 8, *D. submarina* KMM 9415^T^; 9, *D. pacifica* NH131^T^.

*Summed features represent groups of two or three fatty acids that could not be separated by GLC with the MIDI system. Summed feature 3 contained C_*16:1*_ω7c and/or iso-C_*15:0*_ 2-OH.

Metabolic profiling indicated that isolate 17-2-E-8 utilized α-D-glucose, α-D-lactose, maltose, D-mannose, L-arabinose, D-cellobiose, L-fucose, D-galactose, gentiobiose, and melibiose, but not D-fructose, lactulose, or D-sorbitol (**Table 5**). The bacterium thrived in the presence of D, L-lactic, D-gluconic, hydroxybutyricacid, and D-saccharic acids while growth was inhibited in the presence of acetic, citric, formic, malonic, propionic, and succinic acids, respectively.

The MALDI-TOF ribosomal signature failed to reliably match 17-2-E-8 to *Devosia* at the genus level (matching score was lower than the acceptable 1.700 threshold) and the closest hit correlated to *Lactobacillus plantarum* ssp. *plantarum* (with a matching score of 1.399). This finding was not surprising as the representation of the *Devosia* genus within the searched MALDI Library (v.081213) is small with only *D. riboflavina* representing the entire *Devosia* genus.

### Phylogenetic Analysis, Whole-Genome Sequencing, and Strain Comparisons

**FIGURE 6:**
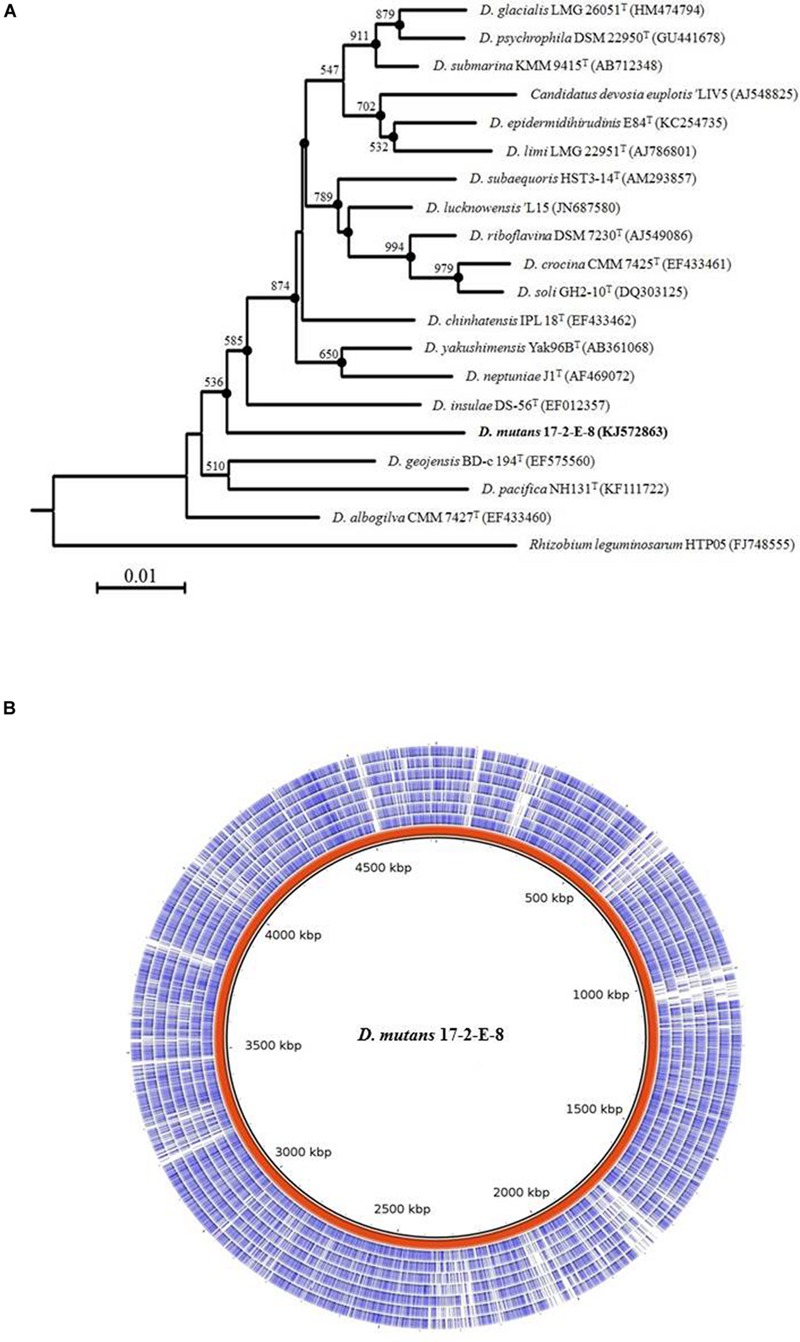
**Isolate 17-2-E-8 represents a new *Devosia* species. (A)** 16S rRNA gene phylogenetic tree reconstruction. The genus *Devosia* currently has sixteen characterized species. There is also one species that is not validated yet. 16S rRNA gene phylogenetic analysis shows how isolate 17-2-E-8 relates to the other species. The tree was generated using neighbor-joining method as described earlier with 1000 bootstraps. Filled circles indicate that the corresponding branches were also recovered in the maximum-parsimony method. Bootstrap resampling values (>50%) are shown with a scale at the bottom reflecting nucleotide substitutions. Bar, 0.01 substitutions per nucleotide position. **(B)** Pair-wise comparisons of multiple genomes of *Devosia* type-strains assembled using the *de novo* approach. The entire genome of *Devosia mutans* 17-2-E-8 (in red) was aligned with *D. geojensis* (DSM19414), *D. psychrophila* (DSM22950), *D. chinhatensis* (DSM24953), *D. soli* (DSM17780), *D. limi* (DSM17137), *D. epidermidihirudinis* (DSM25750), and *D. riboflavin*a (IFO13584) genomes (inner to outer circles, respectively). Nucleotide sequence comparisons were conducted using BRIG. The comparison clearly shows the uniqueness of *Devosia* 17-2-E-8 at the genome level in relation to the closest validated *Devosia* species.

The 16S rRNA gene sequence alignment mapped isolate 17-2-E-8 to other species within the *Devosia* genus with the closest species match to *D. insulae* DS-56^T^at 95% sequence similarity. Multiple sequence alignments (Clustal_W) and the construction of phylogenetic trees with 1000 bootstraps showed how this isolate related to other species within the *Devosia* genus (**Figure 6A**). The results supported clustering 17-2-E-8 with other members of the *Devosia* genus yet forming an independent lineage adjacent to *D. insulae* DS-56T (EF012357). The 16S DNA gene sequence (1421 bp) was deposited within the NCBI nucleotides collection under accession number (KJ572863). The G + C content of isolate 17-2-E-8 was calculated at 63.95%, falling within the range that has been previously reported (59.5 to 66.2%) for the genus *Devosia*. The pair-wise comparisons of multiple genomes of *Devosia* type-strains (**Figure 6B**) assembled using the *de novo* approach clearly confirmed the uniqueness of *Devosia* 17-2-E-8 at the genome level in relation to the closest validated *Devosia* species (**Figure 6B**).

## Discussion and Conclusion

Considering the dynamics of ecological communities, strategies that adopt the enrichment of growth media with natural sources of detoxifying bacteria are more likely to succeed in isolating microorganisms capable of bio-transforming the target toxins (Volkl et al., 2004; Ito et al., 2013). In such cases, it is hypothesized that DON-transforming microbes will be able to grow in environments that contain a high population of the plant pathogen *Fusarium* spp. with high rates of host plants infection. In the present study, a bacterium 17-2-E-8 that can detoxify DON was isolated from soil samples enriched with *F. graminearum*-infested corn for 6 weeks. This approach may also be applied to select microorganisms that transform other natural toxins or environmental contaminants.

The isolated bacterium was confirmed to belong to the *Devosia* genus (Nakagawa et al., 1996; Rivas et al., 2003; Vanparys et al., 2005; Yoo et al., 2006; Lee, 2007; Yoon et al., 2007; Kumar et al., 2008; Ryu et al., 2008; Verma et al., 2009; Bautista et al., 2010; Zhang et al., 2012; Dua et al., 2013; Galatis et al., 2013; Romanenko et al., 2013). The results from 16S rRNA gene sequence similarity and phenotypic characterization support that isolate 17-2-E-8 represents a new species, for which the name *Devosia mutans* (mu’tans, L. part. adj; mutans, pertains to the ability of this species to transform or convert deoxynivalenol) is established, with the type strain 17-2-E-8 (IDAC 040408-1 = ATCC PTA-121309).

The ability of *D. mutans* 17-2-E-8 to grow and transform DON in various media (such as CMB and LB, *etc*) confirmed that this bacterium does not require DON to be the sole source of carbon to commit DON to the bio-transformation pathways, which highlights the possibility for an empirical use of such a strain within the feed industry. Strain 17-2-E-8 plasticity of transforming DON was maintained even with broths rich in other organic source of carbon (such as LB, CMB, peptones and corn steep liquor). This is different from most previously reported isolates such as *Agrobacterium-Rhizobium* sp. strain E3-39 (Shima et al., 1997), *Sphingomonas* sp. strain KSM1(Ito et al., 2013), and *Nocardioides* sp. strain WSN05-2 (Ikunaga et al., 2011) that demand the presence of DON as the sole source of carbon for detoxification. Based on the results presented above, further investigations about the nature of DON detoxification capabilities of *D. mutans* 17-2-E-8 are proposed to explore its full-potential especially in the light of the recently confirmed abrogation of toxicity of 3*-epi*-DON (He et al., 2015a).

Growth and environmental factors can have a strong influence on bacterial cultures and their related functions/phenotypes (von Stetten et al., 1999; Coggan and Wolfgang, 2012). In the current study, we examined the influence of several such factors on the growth of, and DON epimerization by, isolate 17-2-E-8. The presented data showed that the isolated bacterium was involved in transforming DON to 3-*epi*-DON under aerobic conditions and in the presence of oxygen where it maintains optimal growth and functionality. Previously reported isolates originating from animal digestive-systems showed the need of strict anaerobic conditions to survive and transform DON (He et al., 1992; Fuchs et al., 2000, 2002) and, hence, limiting their practical use.

Equally important is that *D. mutans* 17-2-E-8 grows at mild temperature (25–28°C) whereas other bacterial strains isolated from ruminants and poultry guts grow at higher temperature (i.e., 37°C) (He et al., 1992). The results obtained in the present study showed that *D. mutans* 17-2-E-8 is not well adapted to temperature below 20°C, but it grew fairly well in a broad range of temperature spanning 20–40°C. As soil microorganisms are classified into three groups according to temperature tolerances, *D. mutans* 17-2-E-8 should be classified as a mesophilic bacterium. Similarly, DON bio-transformation of *D. mutans* 17-2-E-8 appeared to be very efficient within the range of 20–35°C where the bacterium exhibited the highest DON detoxification activity at 28–30°C. As the temperature increased and while the bacterium retained its growth capacity at 40°C, no DON-bio-transformation was detected at this temperature suggesting a low correlation between the biomass and DON-bio-transformation at such elevated temperatures. These findings are in agreement with the study by Volkl et al. (2004) in which a mixed bacterial culture transformed DON at temperature ranging from 20 to 30°C but the functionality was lost when temperatures were above 37°C.

In a similar fashion, the initial pH of the media had a significant effect on the growth of *D. mutans* 17-2-E-8 and DON bio-transformation. In general, soil bacteria grow optimally at a pH near neutrality (Feng et al., 2008). *D. mutans*17-2-E-8 reached its maximal DON biotransformation activity at pH = 7–8; however, it could not tolerate extreme alkaline or acidic conditions which resulted in minimal/no DON bio-transformation. The effects of pH levels may be attributed to the interference of hydrogen atoms with enzyme-assisted epimerization (Goese et al., 2000).

When individual nitrogen and carbon sources were used in the media, changes in the isolate’s growth and DON bio-transformation were observed regardless of the presence of minerals. In the presence of organic nitrogen sources (yeast extract and peptones), the bacterium grew well while inorganic nitrogen sources (ammonium sulfate and ammonium nitrate) resulted in poor growth. Yeast extract and peptones are well known for supporting growth of microorganisms (Zhang et al., 2011). These substrates not only contain balanced levels of amino acids and peptides but also water-soluble vitamins, minerals, and carbohydrates. Yeast extract and peptones supported the rapid growth of *D. mutans* 17-2-E-8 independent of the supplementation with minerals, however, differences were found in DON bio-transformation capacity when minerals were added to corn steep liquor and peptones media. Peptone-containing media exhibited a small increase while corn steep liquor broth showed a significant improvement in DON biotransformation. This increase in the bio-transformation could be related to particular elements such as Mg^2+^ and Fe^2+^ considered as enzyme cofactors (Boll and Fuchs, 1995). Alternatively, the addition of minerals may also have shifted the pH of the medium drastically increasing DON bio-transformation by *D. mutans* 17-2-E-8 as it was the case in corn starch medium and ammonium sulfate broth (**Table 4**). The inability of urea broth to support DON bio-transformation can be logically explained by the unsupportive pH value (alkaline conditions) observed in this media even in the presence of minerals.

The epimerization function in the above studies correlated positively with the numbers of viable bacterial cells suggesting an enzymatic pathway responsible for the noted activity. It should be noted that most of the enzymatic processes are dependent on the availability of specific co-factors (Lyagin et al., 2011) including metals, the current study clearly demonstrates that mineral mixtures addition substantially enhanced DON bio-transformation capabilities of *D. mutans* 17-2-E-8.

In summary, the optimal growth and DON biotransformation conditions of *D. mutans* 17-2-E-8 include: temperatures close to 28°C, close to neutral pH of 7, and an organic source of nitrogen and carbon in the presence of aerobic atmosphere. Under the aforementioned conditions, 3-*epi*-DON is the primary product of the conversion in a process that is fundamentally enzymatic in nature. The observed accumulation of 3*-epi*-DON within the growth medium keeps the question about the fate of 3-*epi*-DON open for future investigation. Collectively, these conditions and the efficient capacity to detoxify DON (3 μg/h/10^8^ cells) distinguish *D. mutans* 17-2-E-8 from previously reported bacterial isolates. The present research serves as a foundation for development of a feed treatment to detoxify DON in contaminated grains for industrial application, under mild and empirical field conditions particularly in liquid-feeding systems or prior to fermentations with lacto-bacteria.

## Author Contributions

Design of the work: JWH, YH, NP, GB, and TZ. Conducting experiments: JWH, YH, NP, and X-ZL. Interpretation of data: JH, YH, NP, GB, and TZ. Drafting the work: JWH, YH, NP, GB, and TZ. Final approval: JWH, YH, GB, and TZ.

## Conflict of Interest Statement

The authors declare that the research was conducted in the absence of any commercial or financial relationships that could be construed as a potential conflict of interest.

## Footnotes

1 http://imed.med.ucm.es/Tools/sias.html
